# Sialoendoscopy and combined approach for the management of salivary gland stones

**DOI:** 10.1007/s00405-012-2145-x

**Published:** 2012-08-09

**Authors:** Tomasz Kopeć, Witold Szyfter, Małgorzata Wierzbicka

**Affiliations:** ENT Department, K. Marcinkowski Medical University, Przybyszewski Street 49, 60355 Poznan, Poland

**Keywords:** Sialolithiasis, Sialoendoscopy, Surgery, Parotid gland, Combined approach

## Abstract

The introduction of minimally invasive surgical procedures has significantly reduced the rate of major salivary gland removal due to sialolithiasis. The aim of this study is to assess the effectiveness of sialoendoscopy, rate of salivary fistula or natural ostium stenosis in parotid sialolithiasis treatment. The endpoint was to analyse the efficiency of a combined transcutaneous and endoscopic approach in the removal of refractory and impacted stones in most difficult cases. Study Design: prospective study, tertiary university centre, between XII 2008 and XI 2011, 185 sialendoscopies (SE) were performed in 162 patients. Within the group of 29 patients with parotid sialolithiasis endoscopy was the definite treatment in 15 cases (53 %), in 9 cases lithotripsy (ESWL) was necessary and in 5 patients who failed SE and lithotripsy, a combined approach was performed. This approach comprised both SE and open surgery. We observed no salivary fistula formation after the incision of the duct. Stenosis of the natural ostium thanks to the insertion of stent was observed only in one case. Sialoendoscopy is the method of choice with a high rate of success and gland preservation in small and medium stones. The combined transcutaneous and endoscopic approach is indicated for large stones, for complications after and contraindications in using minimally invasive procedures. Short and medium term follow up shows that surgery can be performed with a high rate of success.

## Introduction

Sialolithiasis is the most common cause of inflammatory disease of large salivary glands and occurs in about 1.2 % of the population [9, 18]. It most often occurs in the submandibular gland—(87 %), followed by the parotid gland—(10 %) and the sublingual gland—(3 %). Sialolithiasis, in as many as 70 % of the cases, is the cause of parotid gland swelling [18]. Sialoliths can occur as single or multiple stones of various shapes and sizes. They are distally and proximally located in the efferent duct, but they may also be found intraparenchymally (outside the main tree of secretory ducts). The annual increase in the size of salivary stones is estimated at 1 mm [11].

The introduction of sialendoscopy has significantly reduced the number of salivary glands removed because of salivary gland stones [5, 6, 8, 9, 11, 14]. It is commonly believed that stones of up to 4–5 mm in diameter can be successfully removed through sialoendoscopy. This applies especially to stones which lie freely in the lumen of the duct and are mobile. In these cases, the stones can be extracted under endoscopic control in more than 80 % of the cases [8, 9, 11]. Larger sialoliths may, however, be fragmented in the lumen of the duct, mechanically or using a laser beam.

Laser fragmentation is performed in few centres, with good results (First International Sialendoscopy Conference, Geneva, 24–25 March 2012), but its use must be done cautiously, because of the potential risk of perforation and further stricture because of heating and absorption in the surrounding tissue [21]. In experienced hands–however, with continuous cold saline rinsing and avoiding shooting against the walls, these risks remain minimal.

Another possibility for the fragmentation of large sialoliths is to perform extracorporeal shock wave lithotripsy (ESWL). It allows the fragmentation of stones of any size and location; it is believed, however, that up to three sessions of lithotripsy are required. However, the effect of ultrasound may have the consequences despite beam concentration: possible damage of surrounding tissues. According to published data, the use of lithotripsy is effective in 75 % of the cases, and allows for the complete retrieval of stones in half of the cases [2, 3, 6, 7, 19]. The rate of success for lithotripsy clearly decreases with the increase in stone diameter. Despite notable technological progress, 5–10 % of patients with parotid gland sialolithiasis cannot be successfully treated using minimally invasive techniques. The main cause appears to be the large size of the stones and long-standing history of recurrent inflammations, which leads to the impaction of the sialolith to the wall of the efferent duct. In these cases, an alternative to the complete removal of the gland is the double-approach procedure: external approach, permitting the retrieval of the sialolith and endoscopic access enabling the monitoring of the lumen of the efferent duct. The aim of this paper was to assess the effectiveness of sialoendoscopy, the rate of salivary fistula formation or natural ostium stenosis in patients with parotid sialolithiasis. The endpoint of this analysis was to present the efficiency of a combined transcutaneous and endoscopic approach in removing refractory and impacted stones.

## Materials and methods

In this prospective study, carried out from December 2008 to November 2011 in a tertiary university centre (Department of Otolaryngology and Laryngological Oncology, Poland) 185 sialoendoscopic procedures in 162 patients (105 females and 57 males) were performed. In our findings 141 patients had obstructive pathology of salivary glands, 84 had confirmed gland or duct sialolithiasis, the distribution being 29 for the parotid and 55 for the submandibular gland. The preoperative diagnosis consisted of routine real-time B-mode ultrasonography in all patients; additionally CT was performed in 11 cases. Our research was approved by Bioethical Commission.

During interventional sialoendoscopy 1.3 and 1.6 mm diameter endoscopes (Karl Storz Tutlingen, Germany, compact modular semirigid interventional endoscope with three channels) were used. Stones were removed with the help of the 0.4 mm diameter wire basket and forceps, introduced through the working canal. In the group of these patients, whose stones were removed via the use of an endoscope only, the sialoendoscopy procedure was carried out after premedication (Midazolam, 7.5 mg) in local anaesthesia. ESWL fragmentation was performed using electromagnetic lithotripter Minilith SL1 (Storz Medical, Switzerland) with integrated ultrasound localization. The patients underwent three sessions, no sedation was needed. The intensity of the waves increased from 1,300 to 7,000 pulses.

The combined approach including sialendoscopy and open parotid surgery was performed in general anaesthesia using facial nerve intraoperative monitoring, with nerve monitor leads from three branches of the facial nerve in the areas around the corner of the eye and mouth.

### Description of the procedure

The presence of stones in the lumen of the duct was confirmed by direct visualisation using 1.3 and 1.6 mm diameter endoscopes. The type of skin incision depended on the location of the stone. In two patients with a distal location of the calculi, a horizontal incision along the skin fold of the cheek at the level of the stone was carried out; the duct was identified using endoscopic transillumination. In the other three patients an S-shaped incision in the preauricular region was performed: two had calculi located proximally, and one patient had stenosis of the natural ostium. The skin flap was elevated to the middle of the cheek closely over the stone, to the point previously marked on the skin during preoperative ultrasound; an endoscopic transillumination of the duct was also performed during this procedure.

In the group of 29 patients with parotid sialolithiasis, the age of the patients ranged from 21 to 83 years, the mean being 53 years. The prevalence of comorbidities was diabetes in four cases, cardiac insufficiency in five patients and chronic pulmonary disease in two patients. The duration of complaints ranged from 6 months to 17 years, the mean being 3.1 years.

Although the paper had a predominantly descriptive character, some statistical analysis was performed using Spearman and Kruskal–Wallis tests.

## Results

Comorbidities and patients’ age had no correlation on sialoendosoppy stone removal rates, these were, however found to be statistically dependent–the duration of complaints; a history of more than 5 years doubled the risk of failure (*p* < 0.005).

Out of 29 patients with parotid gland sialolithiasis, stones were removed endoscopically in 15 patients (53 %), including 7 patients who had minipapillotomy performed due to large stone size. In one case, multiple stones (5 sialoliths) were removed. To avoid duct stenosis, a stent was introduced after endoscopy for 28 days. A flexible catheter wit external diameter 1.1–1.3 mm was used as a stent. Such catheter is typically used for vascular radiological examinations. Here, the stent was introduced into the duct at the end of the procedure and then sutured to the mucosa of the vestibule of the mouth. No problems were observed at stent retrieval. In the case of one patient, after the removal of a 12 mm stone located near the ostium of the Stensen’s duct, despite the insertion of a stent, we observed a complete stenosis. This finally led to the dilatation of the duct’s lumen and inflammation of the gland. In 14 cases (47 %) cases endoscopy revealed that the stone was large, had a rough surface, completely obliterated the lumen of the duct and was closely impacted to its wall, thus could not be removed. These patients were referred for lithotripsy: in nine cases, the stone fragments were evacuated; in five cases, evacuation was spontaneous; and in other four cases, sialoendoscopy was used for their evacuation. The evacuation was monitored with the use of ultrasound. Yet, in the case of five patients, the calculi persisted. In these patients due to the lack of improvement, it was decided to carry out a double-approach procedure (external and endoscopic).

The subject of special interest was these five patients who failed the mini invasive approach and demanded more aggressive treatment. They were aged from 46 to 75, mean 62 years. The complains duration ranged from 7 to 17 years, the mean being 12 years, which was significantly longer as compared to the rest of the group (*p* < 0.005). In one patient the stone was located proximally (away from the natural ostium of the duct). In the other two cases, the calculi were located distally, in the soft tissues of the cheek. The fourth patient had a fistula extending from the surface of the cheek leading towards the stone. The fifth patient had a stenosis of the ductal ostium after the removal of a huge stone, which could not be accessed from the oral cavity (Table 1).

**Table 1:** Characteristic of patients treated with combined approach

Age, gender	Indication	Stone localisation	Stent	Current symptoms	Follow-up (months)
K.A. 46, female	Stensen’s duct calculi	Distally, fistula	Yes	None	29
P.T. 75 female	Stensen’s duct calculi	Distally	Yes	None	27
K.E. 66, female	Stensen’s duct calculi	Proximally	Yes	None	23
R.M., 48, female	Ostial stenosis after stone evacuation	State after removal of stone by an incision of mucosa of the cheek	Yes	None	21
L.B., 73 female	Stensen’s duct calculi	Proximally	Yes	None	2

**Fig. 1:**
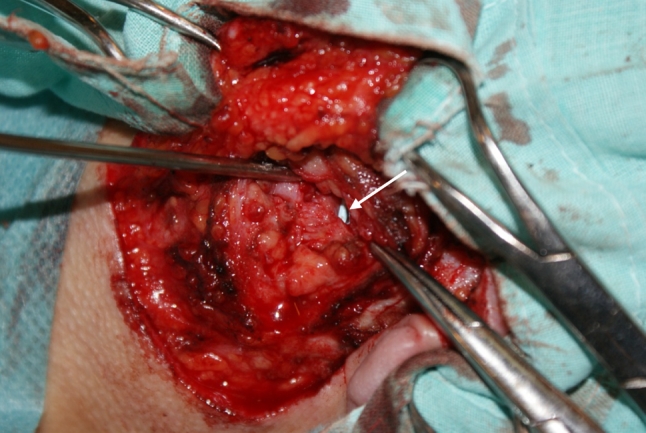
Combined approach. The preauricular flat was elevated. Sialodochotomy was performed and stone was removed under the guidance of an endoscope. A stent was inserted through the papilla to the proximal part of Stensen’s duct (*arrow*). The incision in the wall of the duct was sutured

**Fig. 2:**
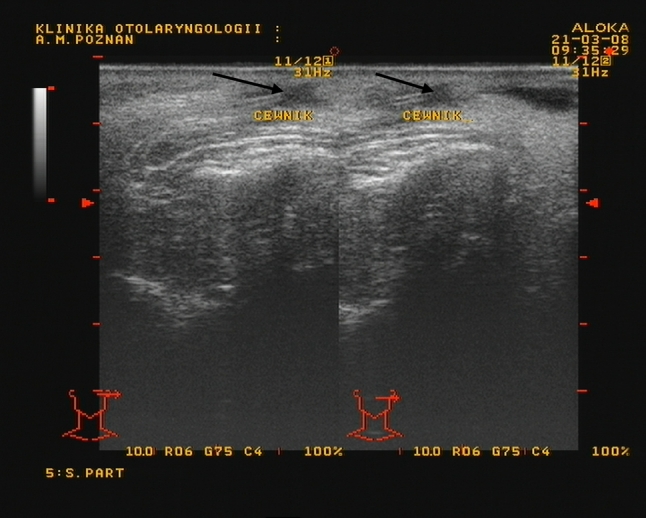
The position of the stent in ultrasound examination (*arrows*)

In four patients with sialolilthiasis, the duct was incised at the level of the stone under the control of a microscope. The sialolith was removed using small forceps. In two cases, a conglomerate of small sialoliths completely filling the lumen of the Stensen’s duct was evacuated. Three stones were removed from a patient with a fistula, one of them was located exactly in the fistula which had to be surgically excised. In one patient with a 17-year history of sialolithiasis, the stone was closely impacted within the wall of the efferent duct. In these four cases stents (stent diameters of 1.1–1.3 mm) were introduced through the natural orifice to avoid duct stenosis (Fig. 1). In the fifth patient with stenosis of the natural orifice, a stent was inserted under the guidance of a sialoendoscope into the lumen of Stensen’s duct to create a new passage to the buccal vestibule. The stent was sutured to the vestibule mucosa and left for 28 days, its position was controlled by ultrasonography (Fig. 2). The perioperative management included the use of antibiotics, pressure dressing for 2–3 days and instructing the patients to avoid food which might cause excessive salivation.

The follow-up period after the operations ranged from 2 to 29 months, mean 20.4. There was no incidence of salivary fistula after the incision of the duct; there was also no stenosis of the natural ostium due to the insertion of the stents. The location of the stent was monitored using an ultrasound examination. In the patient with the cicatrised stenosis of the natural ostium, the newly created ostium functioned normally and no further stenosis occurred. In three patients, the parotid gland function was normal, while in one patient with a 17-year history of sialolithiasis and inflammation, symptoms of glandular atrophy, confirmed by ultrasound, were observed. There were also no signs of facial nerve paralysis. None of our patient needed parotidectomy.

## Discussion

The actual mechanism responsible for the formation of stones is currently unknown. According to Harrison [4], one of the hypothesis suggests that under normal circumstances, microcalculi occur, but are spontaneously washed away and removed through the natural ostium of the gland. Disturbances in the chemical composition of the secreted saliva (dyschylia), as well as the impairment of its outflow due to stenosis and distortion of the duct could cause the deposition of mineral salts and increase in the size of the calculi. A second theory, in turn, implicates the existence of “mucous plugs”, which form the nidus for the formation of calculi. The existence of such a nidus enables the deposition of inorganic substances, contributing in this way to the gradual enlargement of the stone size [11].

The use of endoscopic and minimally invasive techniques allows for a wider preservation of the major salivary glands in cases of sialolithiasis. According to literature data, 80–90 % of patients with parotid gland sialolithiasis can be treated using minimally invasive techniques such as sialendoscopy and ESWL [5, 6, 8, 9, 11, 12, 14, 16]. It should be remembered that stones larger than 6 mm in diameter and impacted in the wall of the duct limit the possibility of using sialendoscopy [8, 9, 11, 12, 16]. After ESWL, larger stones (larger than 8–10 mm in diameter) can be successfully fragmented and then removed using a sialoendoscope.

In our data of over 3 years patients with parotid gland sialolithiasis formed 35 % (29 out of 84). Stones were removed endoscopically in 15 patients, including one case of multiple stones. The success rate of sialendoscopy approached 53 %. In none of the patients parotidectomy was indispensable.

There are few reports in literature concerning the removal of large stones from the parotid gland by means of the double approach procedure. It is estimated that, despite sialendoscopy and ESWL, approximately 10 % of sialoliths cannot be removed endoscopically and will continue to be the cause of recurrent inflammations and swellings of the gland [10]. In this situation, the use of the double approach procedure appears to be optimal and complementary to minimally invasive techniques. The transcutaneous and endoscopic approach seems to be beneficial also in those cases, where ESWL is not available. The transcutaneous removal of stones under ultrasound guidance was described in 1991 by Baumarsh et al. [1]. The authors did not, however, use an endoscope to monitor the incision line of the duct. Nahlieli et al. [17] described 12 patients treated for parotid gland sialolithiasis using an external approach procedure and listed the indications for using this technique: location of the stone in the posterior one third of the Stensen’s duct, small duct diameter, stones larger than 5 mm in diameter with unfavourable/insufficient conditions for sialoendoscopic removal, as well as the presence of intraparenchymal stones; successful removal was achieved in 9/12 patients (75 %); in one patient with multiple stones two third were removed; in 7 out of 12 patients (58 %) the gland functioned normally; there were signs of atrophy in 3 patients [17]. Koch et al. [10] described nine patients in whom the double approach was adopted due to the large size of the stone and failure of a previous treatment. Stones were removed in all the patients; however, total parotidectomy was carried out in one of the patients due to the inability to reconstruct the macerated Stensen’s duct. Walvekar et al. [20] used the double approach procedure in 19 out of 106 patients with sialolithiasis (18 %). Stones with no complications were removed in 90 % of the cases. The authors also recommend this procedure for patients with stenosis of the efferent duct [20]. McGurk described the use of the double approach procedure in eight patients: seven had sialolithiasis and one had stenosis of the duct; stones were removed in all seven patients; in one, however, the laceration of the duct unabled its reconstruction and therefore a ligation was performed. The average size of the stones was 11 mm in diameter. All patients experienced improvement; the salivary gland function was preserved in 75 % of the cases [15]. Marchal described his experience with 37 patients having refractory stones larger than 6 mm in diameter and with stenosis of the duct. Resolution of symptoms occurred in 92 % of the patients; the efferent duct was ligated in three out of four patients in whom the treatment failed [13].

In our material the double approach procedure was performed in five patients with parotid obturation, four with sialolithiasis and one patient with stenosis of the distal portion of the duct after papillotomy. During the follow-up period up to 29 months there was no incidence of salivary fistula or stenosis of the natural ostium after the insertion of a stent. There were also no signs of facial nerve paralysis. None of our patient needed Stensen duct ligation or parotidectomy. In four patients the parotid gland function returned to baseline, while in the patient with a 17-year history of sialolithiasis symptoms of glandular atrophy, confirmed by ultrasound, were observed.

The type of skin incision depends on the location of the stone. A preauricular incision, similar to that used during rhytidectomy, is recommended in cases with proximal location of the stone [13, 15, 17]. In our material, it was used in three patients, two with sialolithiasis and one with ostial stenosis. Incision in the cheek skin fold is recommended for cases in which the stone is impacted in the distal portion of the duct, in our group two patients were operated using this technique. The risk of facial nerve damage is minimal with the use of intraoperative monitoring (13.17). We agree with this opinion and in every case used the monitoring, operating safely without facial nerve palsy.

All the authors point out clearly that in order to stabilise the duct and prevent the formation of a secondary stenosis, it is appropriate to insert a stent [13, 15, 17]. We can confirm these recommendations. In our material, the stents diameters were from 1.1 to 1.3 mm and were retained for an average of 28 days. Stent placement can be monitored using ultrasound, both during surgery and follow-up.

The success rate of the combined approach in our five cases is 100 %, the short and medium follow-up period (average of 20.4 months) does not allow us to draw far-reaching conclusions. The average follow-up period of patients treated with the double approach procedure by the other authors was 10 months, McGurk [15]; 18.9 months, Koch [10] 19 months, Marchal [13].

To conclude, the mainstay of sialolithiasis treatment both in submandibular and parotid gland is sialoendoscopy, contemporarily the first line procedure. In the most complicated cases, in the presence of large refractory stones, impacted in the duct wall and in the presence of complications (e.g. fistula) a double approach procedure is indicated. Long-term data and experience with larger groups of patients are not yet available, nevertheless the combined transcutaneous and endoscopic approach seems to be beneficial in all cases where minimally invasive procedures are contraindicated. Short and medium follow-up periods have shown that this method is safe for the patient, allows the resolution of symptoms while retaining the gland and its function with a high rate of success.
